# Identifying knowledge deficiencies in genetics education among medical students and interns in Saudi Arabia- A cross-sectional study

**DOI:** 10.1186/s12909-024-05782-8

**Published:** 2024-07-19

**Authors:** Abeer F. Zakariyah, Sadin A. Alamri, Manal M. Alzahrani, Aseel A. Alamri, Muhammad A. Khan, Mehenaz A. Hanbazazh

**Affiliations:** 1 Department of Medical Genetics, College of Medicine, University of Jeddah, P.O.Box 80327, Jeddah, Saudi Arabia; 2 College of Medicine, University of Jeddah, Jeddah, Saudi Arabia; 3 College of Medicine, Taibah University, Madinah, Saudi Arabia; 4 Department of Medical Education, College of Medicine, King Saud Bin Abdulaziz University for Health Sciences (KSAU-HS), Jeddah, Saudi Arabia; 5 King Abdullah International Medical Research Centre (KAIMRC), National Guard Health Affairs (NGHA), King Abdulaziz Medical City, 21423 Jeddah, Saudi Arabia; 6 Department of Pathology, College of Medicine, University of Jeddah, P.O.Box 80327, Jeddah, Saudi Arabia

**Keywords:** Medical genetics, Medical students, Genetics knowledge, Cross-section, Saudi Arabia

## Abstract

**Background:**

Understanding genetics is crucial for medical students, particularly in Saudi Arabia, where genetic disorders are prevalent owing to high rates of consanguineous marriages. This knowledge is essential for the early detection, prevention, and management of genetic disorders, and for incorporating medical genetics and genomics into patient care. This study aimed to assess the current state of genetics knowledge among medical students and interns across Saudi Arabia and to identify knowledge gaps in genetics.

**Method:**

A cross-sectional study was conducted between August and September 2023 involving 732 medical students from all regions of Saudi Arabia. The participants completed a validated questionnaire assessing their knowledge of basic genetics, genetic inheritance, genetic testing, and clinical genetics.

**Result:**

Over 60% of medical students and interns reported that they considered themselves to have only slight knowledge in all areas of genetics. The results revealed a general lack of medical genetic understanding among students and interns, particularly regarding genetic inheritance and testing. For genetic inheritance, slight knowledge was found in 65.2% of pre-clinical, 60.1% of clinical, and 53.2% of interns, with significant differences between groups (*p* < 0.001). In genetic testing, 75.4% of pre-clinical, 83.9% of clinical, and 90.6% of interns showed slight knowledge, with significant differences across stages (*p* = 0.021). This study also found that lectures, genetics laboratories, and problem-solving sessions were the preferred resources for learning genetics.

**Conclusion:**

The current study revealed a notable deficiency in the understanding of medical genetics among medical students and interns in Saudi Arabia, particularly regarding genetic inheritance and testing. This is consistent with previous research highlighting the widespread lack of genetics knowledge among medical students. Integrating more comprehensive genetics education, especially during the clinical years, could improve students’ preparedness and confidence in managing genetic disorders. These findings highlight the critical need for curriculum development to equip future physicians with the essential skills for managing genetic disorders.

**Supplementary Information:**

The online version contains supplementary material available at 10.1186/s12909-024-05782-8.

## Background

The first decade of the 21st century was marked by rapid discoveries and groundbreaking technological advancements, which led to significant progress in genetics [1]. This rapid evolution in genetics has led to significant medical advances [2]. Genetic testing has immense potential to reduce the prevalence of genetic disorders through early detection and prevention [3]. The application of genomic testing in clinical care has grown substantially and expanded beyond rare diseases. It also assists in diagnosing and managing conditions such as cancer, prenatal care, neurological disorders, and congenital malformations [4, 5]. Ideally, physicians should incorporate medical genetics and genomics into patient care across all healthcare spectrums [6]. Over the past few years, concerns have been raised regarding the rapid integration of genomic medicine into clinical practice and the ability of the education system to adapt to the evolving demands of this field [7].

Genetics education for medical students is key to providing appropriate care for patients with genetic disorders [8]. Proper genetics education for medical students should cover both clinical concepts and knowledge of the fundamental principles of human genetics [9]. The absence of genetics in clinical training and its diluted identity as a discipline have possible adverse effects because specialized training in clinical genetics may reduce and lower recruitment for this vital field [10]. Most medical schools in Saudi Arabia divide education into three phases. The pre-clinical phase includes the first three years of study, during which students undertake genetic courses as part of their basic medical science curriculum. The clinical phase encompasses the fourth to sixth years, and the final phase is the intern year, highlighting that genetics is primarily taught during the pre-clinical years [11]. A previous study conducted at Princess Nourah Bint Abdulrahman University assessed knowledge of genetics among second- and fourth-year medical students. Their study provided valuable insights and revealed a deficiency in genetics education [11]. However, further assessment of genetics knowledge among medical students and interns in all regions of Saudi Arabia has not yet been conducted.

The high prevalence of genetic disorders in Saudi Arabia, due to the high rate of consanguineous marriages, necessitates the knowledge and skills of physicians for effective treatment and management. Medical students must understand the basic concepts of genetic inheritance and testing, as they typically take only one introductory course in genetics during their early years at university. This could lead to suboptimal learning outcomes if the curriculum does not evolve to match rapid advancements in medical genetics. Therefore, more comprehensive studies are required to assess the knowledge of medical students in all regions of Saudi Arabia. This study aimed to assess the current state of genetics education among medical students and interns across Saudi Arabia by identifying knowledge gaps and areas of deficiency in genetics education that have not been adequately addressed in previous research. To our knowledge, this is the first study to evaluate the understanding of genetics among medical students and interns in Saudi Arabia.

## Methods

### Participants and data collection

All participants were provided with thorough information and informed at the beginning of the questionnaire. The nature and goal of the study were declared and informed consent was obtained from all participants. No personally identifiable information such as names or ID numbers was obtained from the participants. Medical students in all regions of Saudi Arabia were included. Based on an estimate of Saudi Arabia’s medical student population, the sample size was estimated to be 379, with a margin of error of 5%, a confidence level of 95%, and a response distribution of 50%. The questionnaire was created using Google Forms and distributed through various digital platforms, including direct messages, WhatsApp, Telegram, and Twitter. The snowball method was used as the distribution strategy, allowing the questionnaire to be widely distributed to medical students across Saudi Arabia.

### Questionnaire

All participants were provided with a validated self-administered questionnaire with structured questions categorized into seven sections: (1) demographic information such as age, gender, region of Saudi Arabia, education level, and year of study; (2) self-assessment of genetics knowledge; (3) assessment of basic genetics knowledge; (4) assessment of genetic inheritance knowledge; (5) assessment of genetic testing knowledge; (6) assessment of clinical genetics knowledge; and (7) choice of the best resources for learning genetics. The options included (a) teaching-learning methods such as lectures, where an instructor delivers theoretical content to a large audience, and problem-solving sessions, which include active learning and are conducted in small groups; (b) learning resources, including textbooks and general search engines; (c) genetic laboratories. The complete questionnaire is presented in Supplementary Data 1.

Our questionnaire was developed based on a genetics curriculum, including basic genetics, Mendelian and non-Mendelian inheritance, clinical genetics, and genetic testing. The researchers developed 16 questions that were validated by genetics experts and a biostatistician. The questionnaire, developed based on learning goals established for undergraduate genetics courses, was validated by conducting a pilot study with 30 students and medical interns to evaluate its reliability, clarity, and effectiveness. No students or interns who had participated in the pilot tests were included in this study. A reliability test was conducted to validate the questionnaires. Cronbach’s alpha was 0.777. The final questionnaire was administered online using Google Forms.

Students were presented with 16 questions divided into four main sections: basic genetics, genetic inheritance, genetic testing, and clinical genetics. Each main section contained four questions, and students’ levels of knowledge were categorized based on the number of correct answers. Students who answered one question correctly or did not answer correctly were classified as slightly knowledgeable, whereas those who answered two or three questions correctly were considered moderately knowledgeable. Those who answered all four questions correctly were considered knowledgeable. This scoring system facilitated the assessment of students’ understanding of various aspects of genetics.

### Data analysis

All baseline characteristics were descriptive. The data analysis was based on the aim of the study. Descriptive statistics were calculated using the frequency and percentages of categorical variables such as gender, region in Saudi Arabia, type of medical school, year of study, and nationality, with a chi-squared test used to compare these variables. Bar graphs were used to represent the categorical data. Data were entered into MS Excel, and the Statistical Package for Social Sciences (SPSS), version 20.0, was used for data analysis. A p-value less than 0.05 was considered to be statistically significant.

## Results

### Demographic information of the participants

A total of 732 medical students participated in our study; their demographic information is summarized in (Table 1). The participants were predominantly female (59.2%, *n* = 433) and from different regions of Saudi Arabia, with the highest proportions in the eastern (27.9%, *n* = 204) and western (27.3%, *n* = 200) regions. Most students were enrolled in government colleges (84.8%, *n* = 621), while 15.2%, *n* = 111 were from private colleges. Most students were in their clinical years of study (53.0%, *n* = 377), followed by pre-clinical years (31.6%, *n* = 231) and interns (15.4%, *n* = 113).

**Table 1:** Demographic characteristics of the participants in the study

Variables		*n* = 732	%
**Gender**			
	Female	433	59.2
	Male	299	40.8
**Nationality**			
	Saudi	664	90.7
	Non-Saudi	68	9.3
**Region in Saudi Arabia**			
	Eastern Region	204	27.9
	Western Region	200	27.3
	Central Region	161	22
	Southern Region	123	16.8
	Northern Region	44	6
**Medical school**			
	Governmental College	621	84.8
	Private College	111	15.2
**Year of study**			
	Clinical	388	53
	Pre-clinical	231	31.6
	Intern	113	15.4

### Medical students’ self-assessment on genetics knowledge

Medical students were asked to conduct a self-assessment of their knowledge in the four categories. The responses were categorized as slightly knowledgeable, moderately knowledgeable, or knowledgeable. We categorized the medical students’ responses based on their years of study. The self-assessment data is summarized in (Table 2). Results showed that in all categories, more than 50% of respondents considered themselves slightly knowledgeable, with statistically significant differences between the groups.

**Table 2:** Medical students’ self-assessment of genetics knowledge

	Year of study								
	Pre-clinical			Clinical		Intern			*P* value
	*n*= 231		%	*n*=388	%	*n*= 113		%	
**Knowledge of basic genetics**									
Slightly knowledgeable	127	(55)		199	(51.3)	74	(65.5)		**0.001**
Moderately knowledgeable	80	(34.6)		143	(36.9)	26	(23.0)		
Knowledgeable	24	(10.4)		46	(11.9)	13	(11.5)		
**Knowledge of genetic inheritance**									
Slightly knowledgeable	136	(58.8)		195	(50.3)	66	(58.4)		**<0.001**
Moderately knowledgeable	74	(32.0)		149	(38.4)	29	(25.7)		
Knowledgeable	21	(9.1)		44	(11.3)	18	(15.9)		
**Knowledge of genetic testing**									
Slightly knowledgeable	179	(77.5)		256	(65.9)	79	(69.9)		**0.001**
Moderately knowledgeable	42	(18.2)		97	(25.0)	24	(21.2)		
Knowledgeable	10	(4.3)		35	(9.0)	10	(8.8)		
**Knowledge of clinical genetics**									
Slightly knowledgeable	184	(79.7)		271	(69.8)	81	(71.7)		**0.001**
Moderately knowledgeable	36	(15.6)		79	(20.4)	23	(20.4)		
Knowledgeable	11	(4.8)		38	(9.8)	9	(8.0)		

Note: Chi-squared test

### Genetic knowledge of medical students

**Fig. 1:**
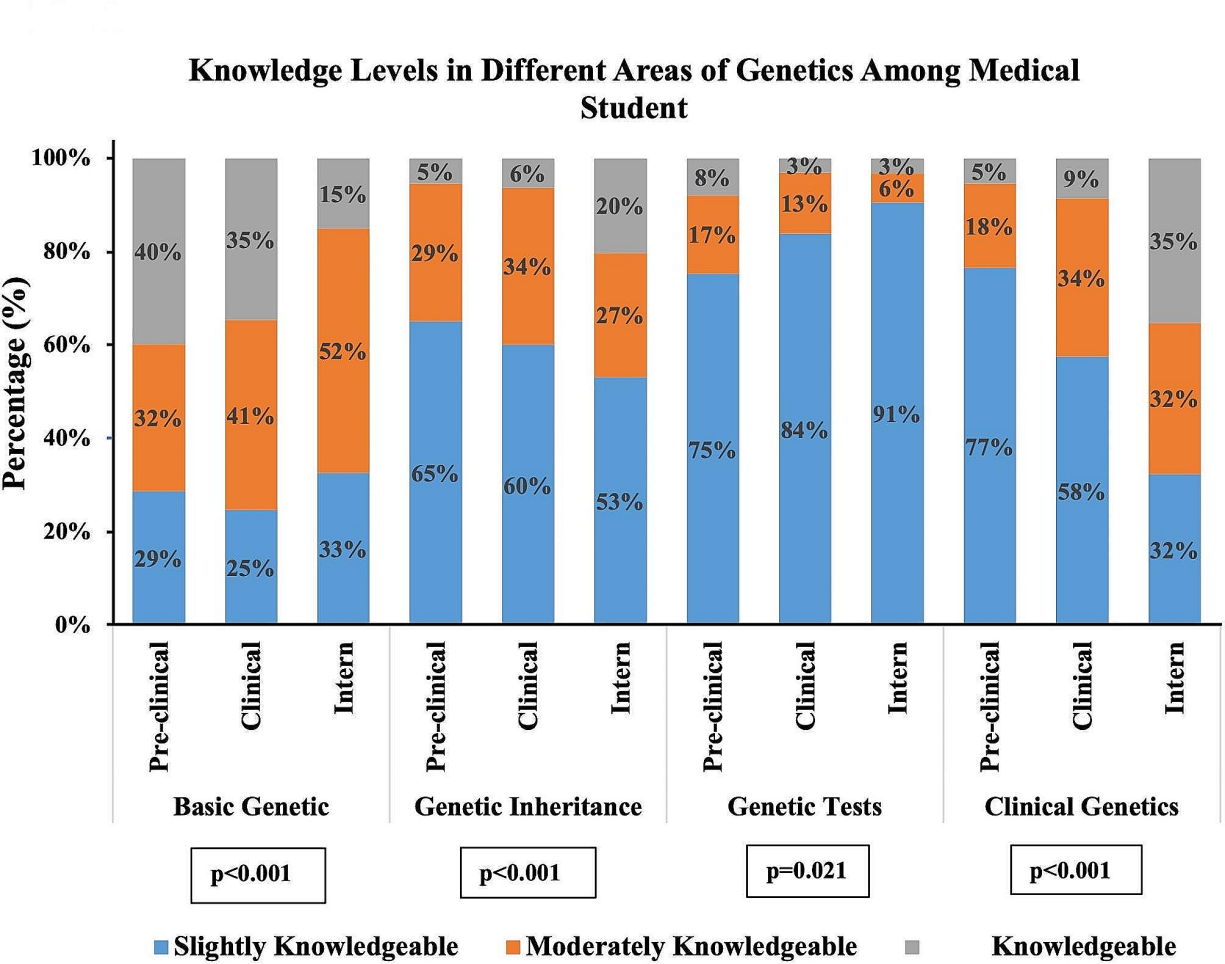
Distribution of knowledge levels in different areas of genetics among pre-clinical students, clinical students, and interns (*n* = 732). The knowledge levels of medical students across four areas of genetics: basic genetics, genetic inheritance, genetic tests, and clinical genetics

The distribution of knowledge levels among the three groups based on years of study is shown in Fig. 1. In basic genetics, the highest percentage of knowledge was observed among pre-clinical students who were knowledgeable (40%; *n* = 87). Among clinical students and interns, the highest percentages were moderately knowledgeable at (40.6%, *n* = 156) and (52.3%, *n* = 56), respectively. There was a significant difference between the groups (*p* < 0.001).

Regarding the genetic inheritance part, the highest percentages of pre-clinical (65.2%, *n* = 120), clinical (60.1%, *n* = 184), and interns (53.2%, *n* = 50) were slightly knowledgeable. Differences in knowledge between these groups were statistically significant (*p* < 0.001).

In the field of genetic testing, the data revealed that most students across all stages of medical education were slightly knowledgeable: pre-clinical (75.4%, *n* = 95), clinical students (83.9%, *n* = 224), and interns (90.6%, *n* = 87) were slightly knowledgeable, with a (*p* = 0.021), indicating a statistically significant difference.

Regarding the clinical genetics section, the group with the highest knowledge was the interns (35.2%, *n* = 38) being knowledgeable, followed by pre-clinical (76.6%, *n* = 141) and clinical students (57.5%, *n* = 202). The differences between the groups were statistically significant (*p* < 0.001).

### Genetic resources preferred by medical students

**Fig. 2:**
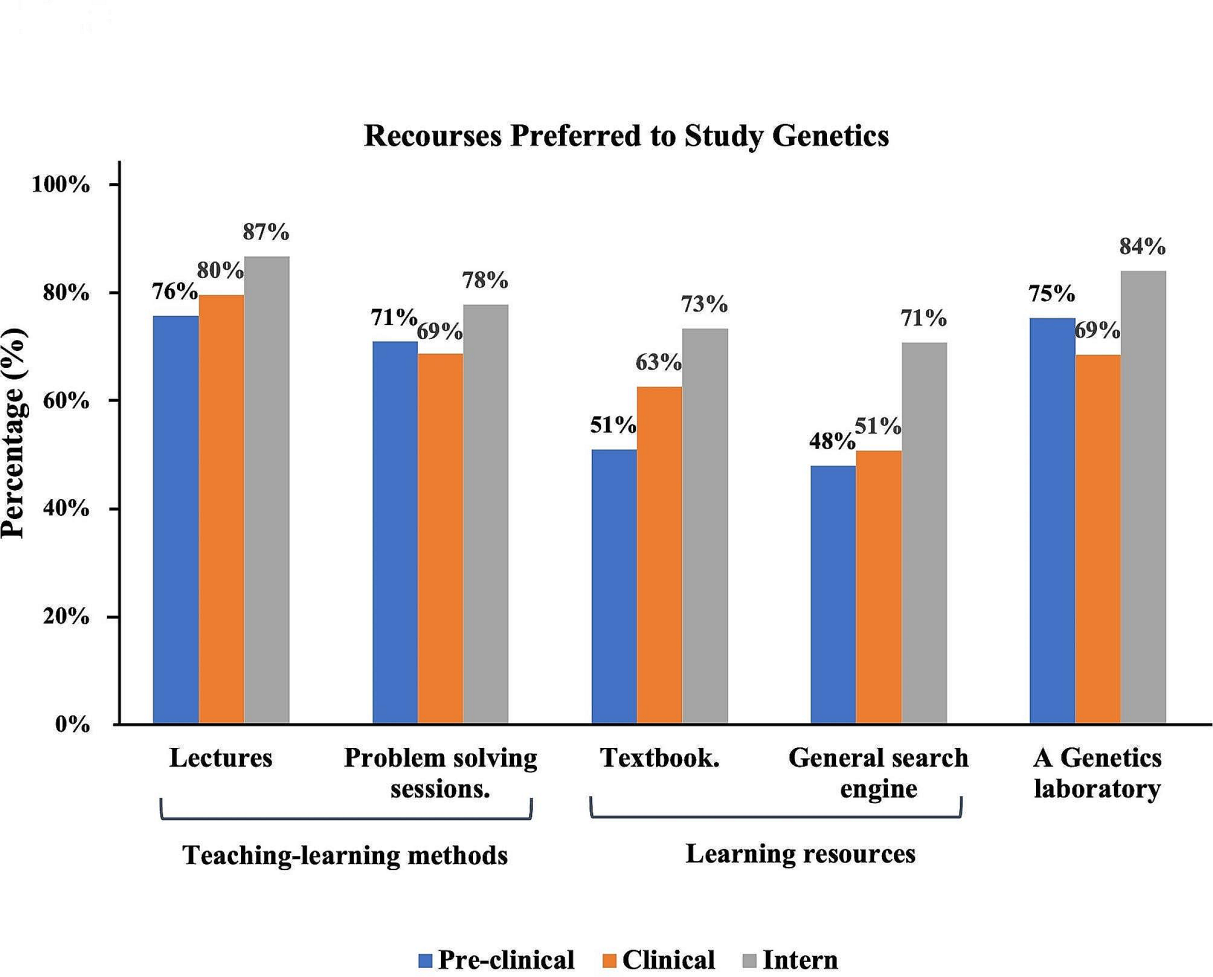
Pre-clinical, clinical, and intern student preferences for genetic learning resources (*n* = 732). The bars show percentages and learning preferences for genetics

We asked the medical students about the most useful resources for learning genetics. Figure 2 shows the number and percentage of students who agreed with, disagreed with, or were unsure about the effectiveness of each resource. The top three resources that all groups agreed were helpful learning resources were lectures (the most agreed-upon), genetics laboratories, and problem-solving sessions. Among interns, (87%, *n* = 98) agreed that lectures were a useful resource, followed by (80%, *n* = 309) of clinical students and (76%, *n* = 175) of pre-clinical students. The genetics laboratory was the second-most agreed-upon resource among interns (84%, *n* = 95), followed by pre-clinical students (75%, *n* = 174) and clinical students (69%, *n* = 267). Problem-solving sessions were the third-most agreed-upon resource among interns (78%, *n* = 88), followed by pre-clinical students (71.0%, *n* = 164) and clinical students (69%, *n* = 267).

## Discussion

Medical genetics is an essential component of clinical practice, and physicians must possess a deep understanding of the principles and practical applications of human genetics. To improve future practice, medical students must thoroughly understand genetics during their undergraduate education to provide more precise diagnoses [12, 13]. Our study assessed genetics knowledge competency among undergraduate medical students at the pre-clinical, clinical, and intern stages across Saudi Arabia. The findings revealed a deficiency in medical genetics knowledge, highlighting significant gaps in the understanding of genetic inheritance and testing among both students and interns. Similar evidence from other studies has shown deficiencies in medical genetics knowledge required for clinical practice [14, 15]. This underscores the need for a comprehensive review and enhancement of the current genetics curriculum to prepare future physicians to manage and treat genetic disorders that are prevalent in the region owing to the high rates of consanguineous marriages [16].

According to our study, the genetic assessment showed that pre-clinical students had the highest scores in basic genetics compared to clinical students and interns. Similarly, a previous study conducted among second and fourth-year medical students showed higher levels of knowledge of molecular and cytogenetics among pre-clinical students than among clinical students [11]. This can be explained by the fact that the educational system of medical schools offers a condensed genetics course in the early years, with a relative lack of genetics content during the clinical years, and a dilution of its identity as a discipline. This could lead to difficulty in recalling the genetic information taught in the initial years [10, 17, 18]. A study conducted on postgraduate pathology residents supports our findings. Year-one residents rated their knowledge of non-genetic topics better than that of genetic topics, with few interactions with genetics professionals. This suggests a deficiency in clinical exposure to geneticists, further emphasizing the need for enhanced genetics education in medical curriculums [18].

Moreover, medical students across all years self-assessed their knowledge of genetic inheritance and testing as slightly knowledgeable, which was consistent with their performance. This finding aligns with those of other studies that have identified gaps in medical students’ understanding of relevant genetic concepts [13, 19]. However, interns demonstrated better knowledge of clinical genetics than pre-clinical and clinical students, likely because of their increased exposure to clinical cases in hospitals. A recent study revealed that most physicians have limited confidence in utilizing medical genetics and genomics in clinical settings, likely due to inadequate training [6]. To effectively apply genetics in medical practice, it is crucial to reinforce this knowledge in clinical settings by connecting molecular genetic mechanisms with clinical features, promoting cognitive integration, enhancing retention and diagnostic accuracy, and ultimately leading to better performance when dealing with complex cases [20]. A previous study found that third-year medical students did not retain the medical genetics knowledge and skills learned in their first year, suggesting the need for a consistent genetics curriculum throughout medical school. [19]. Limited knowledge of genetic testing indicates a curriculum gap, potentially affecting future decision-making and preparedness in ordering and interpreting genetic tests and counseling patients [21].

Medical schools can enhance their learning experiences and achieve better academic outcomes by identifying medical students’ preferred teaching and learning methods. Our study found that over half of medical students preferred lectures, genetic laboratories, and problem-solving sessions for learning genetics, aligning with a previous study among nursing students [22]. The genetics laboratory was the second most favored approach across all three levels, aligning with a study utilizing a virtual genetics laboratory, in which students had improved comprehension of medical genetics and found the experience highly engaging, which increased their interest in the subject [23]. Problem-solving sessions, which included case studies that engaged students with real-life examples, were the third most preferred learning method. A recent study found that case studies effectively teach genetic concepts, such as genomic imprinting, improve students’ test performance, and encourage positive attitudes in the classroom [24]. These sessions also help medical students acquire challenging cognitive skills, preparing them for future clinical practice [25, 26].

The choice of lectures as the preferred method for learning genetics among participants in our study may explain their overall low levels of genetics knowledge. In lectures, the instructor delivers comprehensive theoretical overviews to a large audience, focusing on the delivery of principles and concepts. This suggests the need for more effective teaching methods such as group discussions and problem-solving, which enhance engagement and learning outcomes in genetics education [27]. A multimodal approach combining lectures, laboratory sessions, and problem-solving sessions is recommended for teaching genetics to medical students [28].

This study offers valuable insights into the current state of genetics knowledge among medical students and interns in Saudi Arabia. However, the study has several limitations. This was a cross-sectional study that relied on self-reported data, which could be subject to bias. Furthermore, the study was conducted across different regions of Saudi Arabia, and regional differences in educational practices and resources may have influenced the results. Therefore, the findings may not be generalizable to all medical students and interns in Saudi Arabia or other countries. In addition, the study’s findings may only partially reflect the comprehensive knowledge of genetics among medical students and interns, because the questions cover only a selection of key learning outcomes that medical students are expected to understand.

## Conclusions

The current study assessed the competency of genetic knowledge among medical students and interns across Saudi Arabia, providing insights into the level of student knowledge that could help improve the genetics curriculum. These findings reveal a general need for enhanced medical genetics knowledge, especially in the areas of genetic inheritance and testing.

## Recommendation

Considering the gaps identified in our study regarding the knowledge of genetic inheritance and testing, we recommend an integrated approach that emphasizes these areas. Active learning strategies, such as case-based discussions based on real-world scenarios, should be introduced at the beginning of lectures to foster critical thinking and enhance the understanding of genetic inheritance principles. This approach can be complemented by practical laboratory sessions to bridge the gap between the theory and applications of genetic testing, thereby enhancing students’ understanding of which genetic tests to perform in the future. Furthermore, the curriculum should focus on prevalent genetic disorders in Saudi Arabia, tailoring the content to include the use of genetic testing for early diagnosis, screening, and prevention. Additionally, our findings suggest that incorporating more genetic sessions into the students’ clinical years is advisable to retain genetic information and prepare them for future clinical practice. This is particularly important given the rapid expansion of genomic medicine and increasing need for genetic services. This approach aligns with the national health priorities and aims to reduce the occurrence of genetic diseases in the country.

### Electronic supplementary material

Below is the link to the electronic supplementary material.

Supplementary Material 1

## Data Availability

“The datasets used and/or analysed during the current study available from the corresponding author on reasonable request”.
