# Supplementary material for: Identifying knowledge deficiencies in genetics education among medical students and interns in Saudi Arabia- A cross-sectional study
Source: BMC Med Educ. 2024 Jul 19;24:778. doi: 10.1186/s12909-024-05782-8 (PMC11264939; doi:10.1186/s12909-024-05782-8)
Supplement: Supplementary file 1 — Supplementary Material 1 [file 12909_2024_5782_MOESM1_ESM.docx]

***Identifying Knowledge Deficiencies in Genetics Education Among Medical Students and Interns in Saudi Arabia- A Cross-Sectional Study***

| **Section 1: Demographic information** |
| --- |
|  |
| **Gender:** |
| Female |
| Male |
|  |
| **Nationality:** |
| Saudi |
| Non-Saudi |
|  |
| **Region in Saudi Arabia:** |
| Western region |
| Eastern region |
| Central region |
| Southern region |
| Northern region |
|  |
| **Which of the following best describe your medical school?** |
| Governmental college |
| Private college |
|  |
| **Year of study:** |
| Pre-clinical years (Basic years) |
| Clinical years |
| Intern |
|  |
| **Did you attend genetic lectures during your undergraduate education?** |
| Yes |
| No |

**Section 2: Self-Assessment of Knowledge in Genetics**

**Basic knowledge of Genetics:**

Slightly knowledgeable

Moderately knowledgeable

Knowledgeable

**Basic knowledge of Genetic inheritance:**

Slightly knowledgeable

Moderately knowledgeable

Knowledgeable

**Knowledge of Genetic testing:**

Slightly knowledgeable

Moderately knowledgeable

Knowledgeable

**Knowledge of Clinical Genetics:**

Slightly knowledgeable

Moderately knowledgeable

Knowledgeable

**Section 3: Assessment of Basic Genetics knowledge**

| **1** | **How does RNA differ from DNA?** | | |  |  |  |  |
| --- | --- | --- | --- | --- | --- | --- | --- |
|  | RNA contains Deoxyribose, and DNA contains Ribose. | | | |  |  |  |
|  | RNA contains Uracil and DNA contains Thymine. | | | |  |  |  |
|  | RNA is double stranded, and DNA is single stranded. | | | |  |  |  |
|  | I do not know |  |  |  |  |  |  |
| **2** | **The Central Dogma of Molecular Biology is the flow of Genetic information as following:** | | | | | | |
|  | Replication, Transcription, Translation | | |  |  |  |  |
|  | Translation, Transcription, Replication | | |  |  |  |  |
|  | Transcription, Translation, and Replication | | |  |  |  |  |
|  | I do not know |  |  |  |  |  |  |
| **3** | **Humans have a total of______ Chromosomes.** | | | |  |  |  |
|  | 23 |  |  |  |  |  |  |
|  | 46 |  |  |  |  |  |  |
|  | 48 |  |  |  |  |  |  |
|  | I do not know |  |  |  |  |  |  |
| **4** | **The Different version of a gene is called:** | | |  |  |  |  |
|  | Chromosome |  |  |  |  |  |  |
|  | Loci |  |  |  |  |  |  |
|  | Allele |  |  |  |  |  |  |
|  | I do not know |  |  |  |  |  |  |

| **Section 4: Assessment of Genetic Inheritance knowledge** | | | | | |  |  | | |
| --- | --- | --- | --- | --- | --- | --- | --- | --- | --- |
| **1** | **One couple are both carriers for Sickle Cell Anemia. The risk of transmitting the disorder to their offspring in each pregnancy is:** | | | | | | | | |
|  |  |  |  |  |  |  |  |  |  |
|  | 25% | |  | | |  |  | | |
|  | 50% | |  | | |  |  | | |
|  | 100% | |  | | |  |  | | |
|  | I do not know | |  | | |  |  | | |
| **2** | **A female carrier of Hemophilia A (an X-linked recessive disorder) marries a normal man. The genetic risk (percentage) of their affected male offspring is:** | | | | | | | | |
|  |  |  |  |  |  |  |  |  |  |
|  | 50% | |  | | |  |  | | |
|  | 25% | |  | | |  |  | | |
|  | 100% | |  | | |  |  | | |
|  | I do not know | |  | | |  |  | | |
| **3** | **Males with a _______ are at no risk of transmitting the defect to their offspring.** | | | | | | | | |
|  | Single gene mutation. | | | | |  |  | | |
|  | X-linked gene mutation. | | | | |  |  | | |
|  | Mitochondrial DNA mutations. | | | | | |  | | |
|  | I do not know | |  | | |  |  | | |
| **4** | **The Prader-Willi and Angelman syndromes are examples of:** | | | | | | | | |
|  | Genomic imprinting | | | | |  |  | | |
|  | Mendelian inheritance | | | | |  |  | | |
|  | Chromosomal numerical abnormalities | | | | | |  | | |
|  | I do not know | |  | | |  |  | | |
| **Section 5: Assessment of Genetic testing knowledge** | | | | | |  |  | | |
| **1** | **The most effective method for detecting a point mutation in a monogenetic disease is:** | | | | | | | | |
|  | Direct gene sequencing | | | | |  |  | | |
|  | Fluorescence in situ hybridisation (FISH) | | | | | |  | | |
|  | Southern blot | |  | | |  |  | | |
|  | I do not know | |  | | |  |  | | |
| **2** | **The most effective method for identifying unknown genetic mutations is:** | | | | | | | | |
|  | Next Generation Sequencing (NGS). | | | | | |  | | |
|  | Fluorescence in situ hybridisation (FISH) | | | | | |  | | |
|  | Direct gene sequencing | | | | |  |  | | |
|  | I do not know | |  | | |  |  | | |
| **3** | **The most commonly used technique for detecting and diagnosing the BCR/ABL translocation in patients with Chronic Myeloid Leukemia (CML) is:** | | | | | | | | |
|  |  |  |  |  |  |  |  |  |  |
|  | Fluorescence in situ hybridization (FISH) | | | | | |  | | |
|  | Polymerase chain reaction (PCR) | | | | | |  | | |
|  | Whole exome sequencing (WES) | | | | |  |  | | |
|  | I do not know | |  | | |  |  | | |
| 4 | **The most commonly used genetic test for detecting numerical abnormalities in the fetus is:** | | | | | | | | |
|  | Noninvasive prenatal testing (NIPT) | | | | | |  | | |
|  | Whole genome sequencing (WGS) | | | | |  |  | | |
|  | Fluorescence in situ hybridization (FISH) | | | | | |  | | |
|  | I do not know | |  | | |  |  | | |
| **Section 6: Assessment of Clinical Genetics knowledge** | | | | | |  |  | | |
| **1** | **A female with Edward Syndrome has the following karyotype:** | | | | | | | | |
|  | 46, XX,+18 | |  | | |  |  | | |
|  | 47, XX, +18 | |  | | |  |  | | |
|  | 47, XY, +18 | |  | | |  |  | | |
|  | I do not know | |  | | |  |  | | |
| **2** | **The most likely diagnosis for a newborn with a Triangle-shaped face, webbed posterior neck, Low posterior hairline, posteriorly rotated external ears, and bilateral feet edema is:** | | | | | | | | |
|  |  |  |  |  |  |  |  |  |  |
|  | Klinefelter syndrome | | | | |  |  | | |
|  | Turner Syndrome | | | | |  |  | | |
|  | Down syndrome | | | | |  |  | | |
|  | I do not know | |  | | |  |  | | |
| **3** | **The most common clinical feature of Trisomy 21 is:** | | | | | | | | |
|  | Hypothyroidism | | | | |  |  | | |
|  | Hypotonia | |  | | |  |  | | |
|  | Hypertonia | |  | | |  |  | | |
|  | I do not know | |  | | |  |  | | |
| **4** | **One potentially key element in the development of familial breast cancer is:** | | | | | | | | |
|  | The mutation in BRCA1 & BRCA2 genes | | | | | |  | | |
|  | The mutation in HPC1 and HPC2 genes | | | | | |  | | |
|  | The mutation in APC gene | | | | |  |  | | |
|  | I do not know | |  | | |  |  | | |
| **Section 7: The best resources for learning genetics are:** | | | | | | | |  |  |
|  | 1. **Teaching-learning methods**  - Lectures | | |  |  | | |  |  |
|  | - Problem solving sessions.  1. **Learning resources** | | | |  | | |  |  |
|  | - Textbook | | |  |  | | |  |  |
|  | - General search engine | | | |  | | |  |  |
|  | 1. A Genetics laboratory | | | |  | | |  |  |
|  |  |  |  |  |  |  |  |  |  |
|  |  |  |  |  |  |  |  |  |  |
